# Representational Disparities in the Enrollment of Parkinson's Disease Clinical Trials

**DOI:** 10.1002/mdc3.70009

**Published:** 2025-02-17

**Authors:** Chia‐Chen Tsai, Brendan Tao, Vallen Lin, Jaden Lo, Suhangi Brahmbhatt, Carmen Kalo, Connie Marras, Faisal Khosa

**Affiliations:** ^1^ Faculty of Medicine, University of British Columbia Vancouver British Columbia Canada; ^2^ Faculty of Health Sciences, McMaster University Hamilton Ontario Canada; ^3^ Temerty Faculty of Medicine, University of Toronto Toronto British Columbia Canada; ^4^ Faculty of Engineering, McMaster University Hamilton Ontario Canada; ^5^ Division of Neurology University of Toronto Toronto Ontario Canada; ^6^ Department of Radiology Vancouver General Hospital Vancouver British Columbia Canada

**Keywords:** Parkinson's disease, sex, race, disparity, clinical trials

Parkinson's disease (PD) has diverse clinical presentations across demographics.^1^ Inclusive clinical trial enrollment ensures representative findings, leading to applicable population‐based health decisions. We evaluated the sex, racial, and ethnic representation of PD clinical trial participants relative to the global demographic distribution of PD.

“Parkinson's disease” was searched on ClinicalTrials.gov (02/2000–03/2023) for randomized interventional PD trials (Fig. S1). Reviewers screened trial eligibility and extracted data in duplicate. Each trial's geographic, temporal and age‐specific female prevalence was attained from the Global Burden of Disease (GBD) database. Participation‐to‐prevalence ratios (PPRs) of 0.8–1.2 indicated that the proportion of female trial participants reflects that of the wider population.^2^ Kruskal‐Wallis tests with post‐hoc comparisons (pairwise Mann–Whitney *U* test and Bonferroni continuity correction) were conducted.

Of 328 trials (Table 1), 38.8% were female. The aggregated female PPR was 0.83 (95% CI:0.79–0.86). Among participants in US‐conducted trials, 2.1% were Black or African American, 0.10% American Indian or Alaskan Native (AIAN), 0.22% Native Hawaiian or Other Pacific Islander (NHOPI), and 6.8% Hispanic. While GBD does not report racial or ethnic prevalences, these proportions lag behind the 2021 US demographic representation: 13.6% Black, 1.3% AIAN, 0.3% NHOPI, and 18.9% Hispanic.^3^

**TABLE 1:** Trial characteristics and enrollment of 328 Parkinson's Disease clinical trials by sex, race, and ethnicity

	Trial participant characteristics											
	Total (*N*)	Female: *N* (%)	PPR_sex_: mean (95% CI)	Reported race (*N*)	White: *N* (%)	Asian: *N* (%)	Black or African American: *N* (%)	AIAN: *N* (%)	NHOPI: *N* (%)	Mixed race: *N* (%)	Reported ethnicity (*N*)	Hispanic: *N* (%)^a^
**Overall**	50,448	19,558 (38.8)	0.83 (0.79–0.86)	16,905	14,276 (84.5)	2391 (14.1)	184 (1.1)	23 (0.14)	14 (0.08)	17 (0.10)	12,843	643 (5.0)
**Blinding**												
Double	45,757	17,845 (39.0)	0.84 (0.79–0.88)	15,604	13,015 (83.4)	2375 (15.2)	165 (1.1)	23 (0.15)	14 (0.09)	12 (0.08)	11,548	629 (5.5)
Triple	1182	444 (37.6)	0.76 (0.61–0.91)	812	806 (99.3)	2 (0.25)	2 (0.25)	0 (0)	0 (0)	2 (0.25)	802	4 (0.50)
Quadruple	3509	1269 (36.2)	0.78 (0.66–0.90)	489	455 (93.1)	14 (2.9)	17 (3.5)	0 (0)	0 (0)	3 (0.61)	493	10 (2.0)
*p*‐value	NA	**<0.01** ^b^	0.33	NA	0.09	**0.03** ^i^	0.09	0.16	0.27	0.92	NA	**0.02**
**Phase**												
I	1576	521 (38.9)	0.79 (0.57–1.00)	47,345	454 (71.7)	155 (24.5)	20 (3.2)	3 (0.47)	1 (0.16)	0 (0)	446	39 (8.7)
II	11,975	4568 (38.2)	0.82 (0.76–0.87)	41,396	4200 (96.2)	80 (1.8)	72 (1.7)	4 (0.09)	3 (0.07)	6 (0.14)	4082	285 (7.0)
III	29,350	11,521 (39.3)	0.87 (0.81–0.93)	29,355	7683 (78.2)	2055 (20.9)	60 (0.61)	14 (0.14)	4 (0.04)	5 (0.05)	6479	175 (2.7)
IV	4342	1773 (40.8)	0.85 (0.76–0.93)	4364	1452 (95.8)	31 (2.0)	25 (1.7)	1 (0.07)	4 (0.26)	3 (0.20)	1446	127 (8.8)
NA	3205	1175 (36.7)	0.79 (0.70–0.88)	3473	487 (85.4)	70 (12.3)	7 (1.2)	1 (0.18)	2 (0.35)	3 (0.53)	390	17 (4.4)
*p*‐value	NA	**<0.01** ^c^	0.35	NA	**<0.01** ^g^	**<0.01** ^j^	0.09	**0.02** ^m^	0.33	0.57	NA	0.4
**Study country**												
United States	9027	3193 (35.4)	0.81 (0.74–0.87)	9152	3939 (95.7)	67 (1.6)	87 (2.1)	4 (0.10)	9 (0.22)	12 (0.29)	3503	237 (6.8)
International	19,360	7996 (41.3)	0.83 (0.77–0.89)	19,600	2497 (52.8)	2216 (46.8)	15 (0.32)	4 (0.08)	0 (0)	1 (0.02)	2885	54 (1.9)
Mixed	22,061	8369 (37.9)	0.85 (0.80–0.90)	22,066	7840 (97.3)	108 (1.3)	82 (1.0)	15 (0.19)	5 (0.06)	4 (0.05)	6455	352 (5.5)
*p*‐value	NA	**<0.01** ^d^	0.43	NA	**<0.01** ^h^	**<0.01** ^k^	**<0.01** ^l^	**<0.01** ^n^	0.22	0.75	NA	**0.02** ^o^
**Sponsor country**												
United States	19,589	7120 (36.4)	0.81 (0.76–0.86)	19,719	9227 (91.6)	663 (6.6)	139 (1.4)	17 (0.17)	14 (0.14)	13 (0.13)	8598	498 (5.8)
International	30,859	12,438 (40.3)	0.84 (0.78–0.89)	31,099	5049 (73.9)	1728 (25.3)	45 (0.66)	6 (0.09)	0 (0)	4 (0.06)	4245	145 (3.4)
*p*‐value	NA	**<0.01**	0.24	NA	0.78	**<0.01**	0.64	0.85	**0.02**	0.88	NA	**0.01**
**Start year**												
Before 2009	23,528	8897 (37.8)	0.78 (0.70–0.85)	6296	4576 (80.0)	1120 (19.6)	17 (0.30)	2 (0.03)	2 (0.03)	3 (0.05)	3725	136 (3.7)
2009–2014	18,106	7380 (40.8)	0.88 (0.82–0.94)	31,621	4959 (86.9)	646 (11.3)	81 (1.4)	10 (0.18)	7 (0.12)	6 (0.11)	4043	233 (5.8)
2015–2022	8814	3281 (37.2)	0.81 (0.75–0.87)	18,984	4741 (86.6)	625 (11.4)	86 (1.6)	11 (0.20)	5 (0.09)	8 (0.15)	5075	274 (5.4)
*p*‐value	NA	**<0.01** ^e^	**0.03** ^f^	NA	0.07	0.91	0.62	0.93	0.81	0.66	NA	0.61
**Intervention type**												
Drug	46,210	17,956 (38.9)	0.81 (0.77–0.86)	46,312	13,772 (84.7)	2269 (14.0)	176 (1.1)	22 (0.14)	12 (0.07)	14 (0.09)	12,381	625 (5.1)
Device/Procedure	4238	1602 (37.8)	0.87 (0.78–0.95)	4506	504 (78.8)	122 (19.1)	8 (1.3)	1 (0.16)	2 (0.31)	3 (0.47)	462	18 (3.9)
*p*‐value	NA	**<0.01**	0.60	NA	**<0.01**	0.87	0.2	0.46	0.79	0.97	NA	0.36

Note: *p*‐values <0.05 are in **bold**. International countries include countries in Africa, Asia, Australia, Europe, Middle East, North America, and/or South America.

Abbreviations: AIAN, American Indian or Alaskan Native; CI, confidence interval; NA, not applicable; NHOPI, Native Hawaiian or Other Pacific Islander; NR, not reported; PPR, participation‐to‐prevalence ratio.

^a^ Percentages were calculated as a portion of participants who reported ethnicity.

^b^ *p*‐value for the difference between double versus triple and double vs. quadruple blinding.

^c^ *p*‐value for the difference between phase I vs. II, I vs. III, I vs. IV, II vs. III, II vs. IV, III vs. IV, III vs. NA, and IV vs. NA.

^d^ *p*‐value for the difference between study country US vs. international and international vs. mixed.

^e^ *p*‐value for the difference between start year before 2009 vs. 2015–2022 and 2009–2014 vs. 2015–2022.

^f^ *p*‐value for the difference between start year before <2009 vs. 2009–2014.

^g^ *p*‐value for the difference between phase I vs. II, I vs. III, I vs. IV, II vs. III, II vs. NA, III vs. NA, and IV vs. NA.

^h^ *p*‐value for the difference between study country US vs. mixed and international vs. mixed.

^i^ *p*‐value for the difference between double vs. triple blinding.

^j^ *p*‐value for the difference between phase II vs. III, III vs. IV, and III vs. NA.

^k^ *p*‐value for the difference between study country US vs. international, US vs. mixed, and internation vs. mixed.

^l^ *p*‐value for the difference between study country US vs. international and international vs. mixed.

^m^ *p*‐value for the difference between phase II vs. III.

^n^ *p*‐value for the difference between study country US vs. international.

^o^ *p*‐value for the difference between study country US vs. international, US vs. mixed, and international vs. mixed.

Across PD trials, female enrollment reflected the PD disease burden of the wider population, enhancing trial generalizability and external validity. Adequate female representation may aid in understanding how PD differentially affects sexes and determining the sex‐specific efficacy of investigational drugs. For instance, sex‐specific differences (eg, genetics, hormones, lifestyle) have been suggested to affect PD susceptibility and severity, with estrogen postulated to be neuroprotective.^1^

Conversely, several racial and ethnic groups were underrepresented (Black, AIAN, NHOPI, Hispanic). While PD prevalence is reportedly higher in White populations, Black individuals with PD experience greater motor impairment and mortality rates than White individuals (income‐ and education‐adjusted).^4^ To address this, we recommend mandating race and ethnicity reporting in clinical trials and setting enrollment quotas that reflect the study country's demographic distribution. This underrepresentation mirrors broader healthcare access inequities, as underrepresented groups face systemic barriers (eg, socioeconomic challenges, limited access to specialized care, mistrust of healthcare), limiting trial participation. Efforts to address systemic inequities are needed to ensure equitable representation.

Our study was limited by uncertainties in GBD prevalence estimates. As GBD does not report racial and ethnic prevalences, we were unable to assess PPRs and region‐specific variations. Other limitations include binary sex classifications, exclusion of other demographic data (eg, gender), and the predominance of male‐led trials. Future trials could collect gender data and expand racial and ethnic categories. Trials could recruit in diverse regions, partner with local organizations to build trust, reduce participation barriers (eg, remote participation, travel support), diversify leadership, and provide cultural competency training to address recruitment biases.

Assessing female representation in PD trials relative to the wider PD population remains challenging due to the underdiagnosis of PD in females.^5^ Additionally, racial and ethnic minority groups are underrepresented. Diverse enrollment and equitable healthcare access are needed to further PD knowledge and treatment and inform policy decisions applicable to the broader population.

## Author Roles

(1) Research project: A. Conception, B. Organization, C. Execution; (2) Statistical Analysis: A. Design, B. Execution, C. Review and Critique; (3) Manuscript Preparation: A. Writing of the first draft, B. Review and Critique

C.T.: 1A, 1B, 1C, 2C, 3A.

B.T.: 1A, 1B, 1C, 2A, 2B, 3B.

V.L.: 1C, 3B.

J.L.: 1C, 3B.

S.B.: 1C, 3B.

C.K.: 1C, 3B.

C.M.: 1B, 3B.

F.K.: 1A, 3B.

## Disclosure

**Funding Sources and Conflict of Interest:** No specific funding was received for this work. The authors declare that there are no conflicts of interest relevant to this work.

**Financial Disclosures for Previous 12 Months:** CM received research funding from the Parkinson's Foundation, Michael J. Fox Foundation, International Parkinson and Movement Disorders Society, the Weston Brain Institute and the Mayvon Foundation. FK is the recipient of the Vancouver Medical, Mental and Allied Staff Association Scientific Achievement Award (2024) and the Michael Smith Health Research BC Health Professional‐Investigator award (2023–2028). All other authors have no financial disclosures for the preceding 12 months.

**Ethical Compliance Statement:** Ethics approval and informed patient consent were not necessary for this work as all data are publicly available and no patient data were used. We confirm that we have read the Journal's position on issues involved in ethical publication and affirm that this work is consistent with those guidelines.

## Supporting information


**Figure S1.** Flow diagram of study methods and selection of Parkinson's disease clinical trials for inclusion. ^a^Exclusion criteria included studies that were non‐randomized, non‐interventional, and/or of the wrong condition. ^b^Exclusion criteria included studies that had no results or publications available (*n* = 309), no report of demographic information (*n* = 108), and/or enrolled fewer than 10 participants (*n* = 33). NCT, National Clinical Trial.

## Data Availability

The data that support the findings of this study are available from the corresponding author upon reasonable request.
