# Supplementary figures and images for: Representational Disparities in the Enrollment of Parkinson's Disease Clinical Trials
Source: Mov Disord Clin Pract. 2025 Feb 17;12(6):878–81. doi: 10.1002/mdc3.70009 (PMC12187980; doi:10.1002/mdc3.70009)

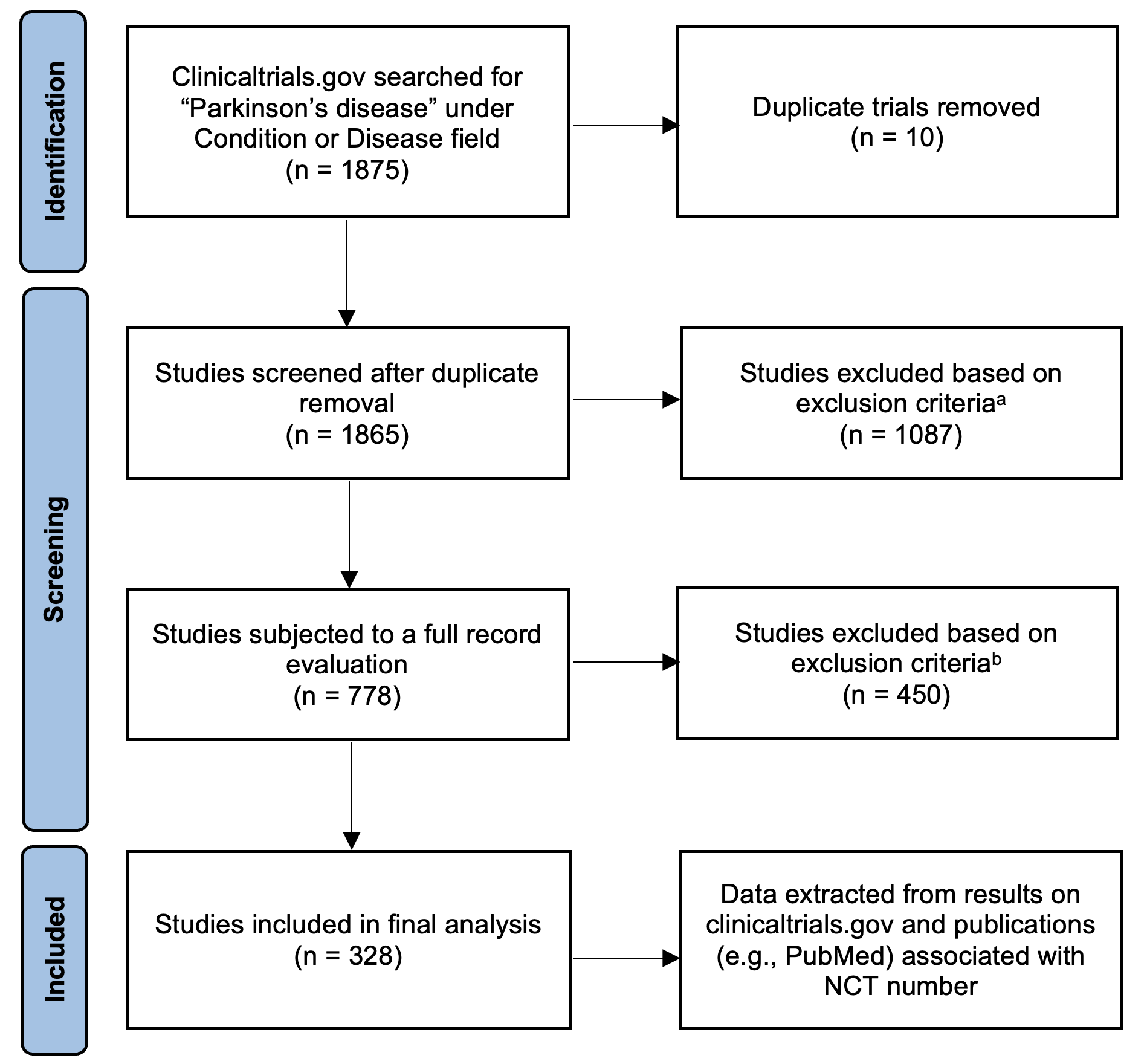

Supplement: Supplementary file 1 — Figure S1. Flow diagram of study methods and selection of Parkinson's disease clinical trials for inclusion. aExclusion criteria included studies that were non‐randomized, non‐interventional, and/or of the wrong condition. bExclusion criteria included studies that had no results or publications available (n = 309), no report of demographic information (n = 108), and/or enrolled fewer than 10 participants (n = 33). NCT, National Clinical Trial. [file MDC3-12-878-s001.png]
